# The risk of infective endocarditis according to blood pressure in patients with diabetes: a nationwide population-based study

**DOI:** 10.1186/s40885-024-00295-4

**Published:** 2024-12-01

**Authors:** Won Kyung Pyo, Hee-Jung Kim, Kyungdo Han, Jin Nam Kim, Se Ju Lee, Jung Ho Kim, Nam Su Ku, Seung Hyun Lee

**Affiliations:** 1 Department of Thoracic and Cardiovascular Surgery, Severance Hospital, Yonsei University College of Medicine, 50-1, Yonsei-ro, Seodaemun-gu, Seoul, 03722 Republic of Korea; 2 Department of Thoracic and Cardiovascular Surgery, Korea University College of Medicine, 222, Korea-daero, Seongbuk-gu, Seoul, 02841 Republic of Korea; 3 Department of Internal Medicine, Severance Hospital, Yonsei University College of Medicine, Seoul, Korea; 4 Department of Statistics and Actuarial Science, Soongsil University, Seoul, Korea; 5 Division of Infectious Disease, Department of Internal Medicine, Inha University College of Medicine, Incheon, Korea

**Keywords:** Infective endocarditis, Diabetes mellitus, Hypertension, Underweight

## Abstract

**Background:**

We aimed to assess the incidence of infective endocarditis (IE) and evaluate the impact of hypertension (HTN) with underweight on the risk of IE among patients with diabetic mellitus (DM) using a nationwide population-based cohort in Korea.

**Methods:**

We identified 2,603,012 participants (57.4 ± 12.3 years) in the national health insurance database. Of these, 374,586 were normotensive, 750,006 were at pre-HTN status, and the remainder had HTN. The risk of IE was compared between the groups, and the impact of being underweight (body mass index < 18.5) was also evaluated.

**Results:**

During follow-up (7.14 years; interquartile range 6.01–8.08 years), 1,703 cases of IE occurred; 168 (0.0647 person per 1000 person-years [PY]), 303 (0.05836 per 1000 PY), and 1,232 (0.12235 per 1000 PY) in normotensive, pre-HTN and HTN group, respectively. Hypertensive participants presented a higher risk of IE (subdistribution hazard ratio, 1.360; 95% confidence interval, 1.152–1.607) than normotensive participants. Being underweight increased the risk of IE by 90% among subjects with HTN. In subgroup analysis, age, duration of DM, insulin use, and habitual factors were not associated with the incidence of IE.

**Conclusions:**

Diabetic patients may require rigorous blood pressure control and simultaneous avoidance of excessive weight loss to prevent IE.

**Graphical abstract:**

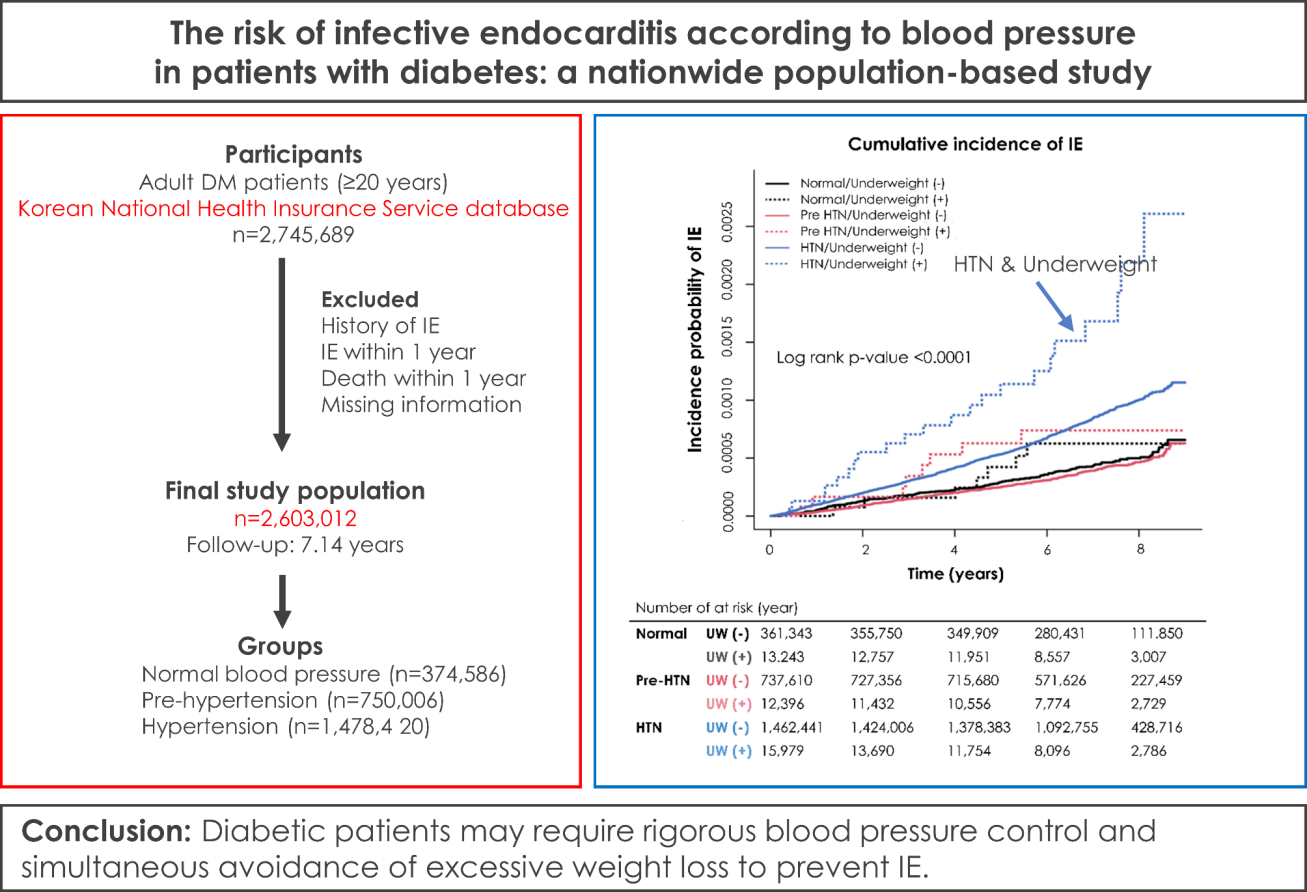

## Background

Despite improvement in diagnostic strategy, antimicrobial treatments, and surgical interventions, infective endocarditis (IE) remains a highly morbid medical condition with substantial mortality and morbidity [1–3]. The risk of incident IE was comparatively well understood in specific high-risk groups including patients with prosthetic valves, intracardiac devices, or heart valve disease [4–6]. Although the epidemiology of IE has been changed from young with rheumatic or congenital valve disease to old with various comorbidities over the last years, little attention has been given to people with diabetes mellitus (DM) who are at risk for various infections.

Previous studies demonstrated that people with DM present a higher susceptibility to infectious disease compared to those without DM, probably due to immune dysfunction, micro-or macro-angiopathy, neuropathy, and consequently increased medical intervention [7–10]. In addition, there is an increasing body of evidence suggesting metabolic diseases such as hypertension (HTN) and being underweight may predispose the risk of infection, and poor clinical outcomes of infectious disease in the underweight population were supported by the ‘obesity paradox’ [11–16]. It implies that HTN and underweight may play a role as a risk factor for incident IE. However, given the paucity of epidemiologic studies, the relationship between the abovementioned comorbidities and incident IE, especially in the diabetic population, remains elusive. Therefore, in the present study, we aimed to investigate the impact of HTN and underweight on incident IE in the diabetic cohort using a nationwide population-based database.

## Methods

### Databases

The present study analyzed the Korean National Health Insurance Service (K-NHIS) and claims database. K-NHIS, a single mandatory insurer managed by the government, covers 97.1% of the Korean population and provides a biennial national health check-up program including questionnaires regarding lifestyle behavior, anthropometric measurements, and laboratory tests. K-NHIS database contains baseline demographic data, diagnostic codes (ICD-10), clinical usage data, medical claim data, and mortality data. The institutional review board waived the need for consent from individual patients because the data used are public and anonymized under confidentiality guidelines.

### Participants

**Fig. 1:**
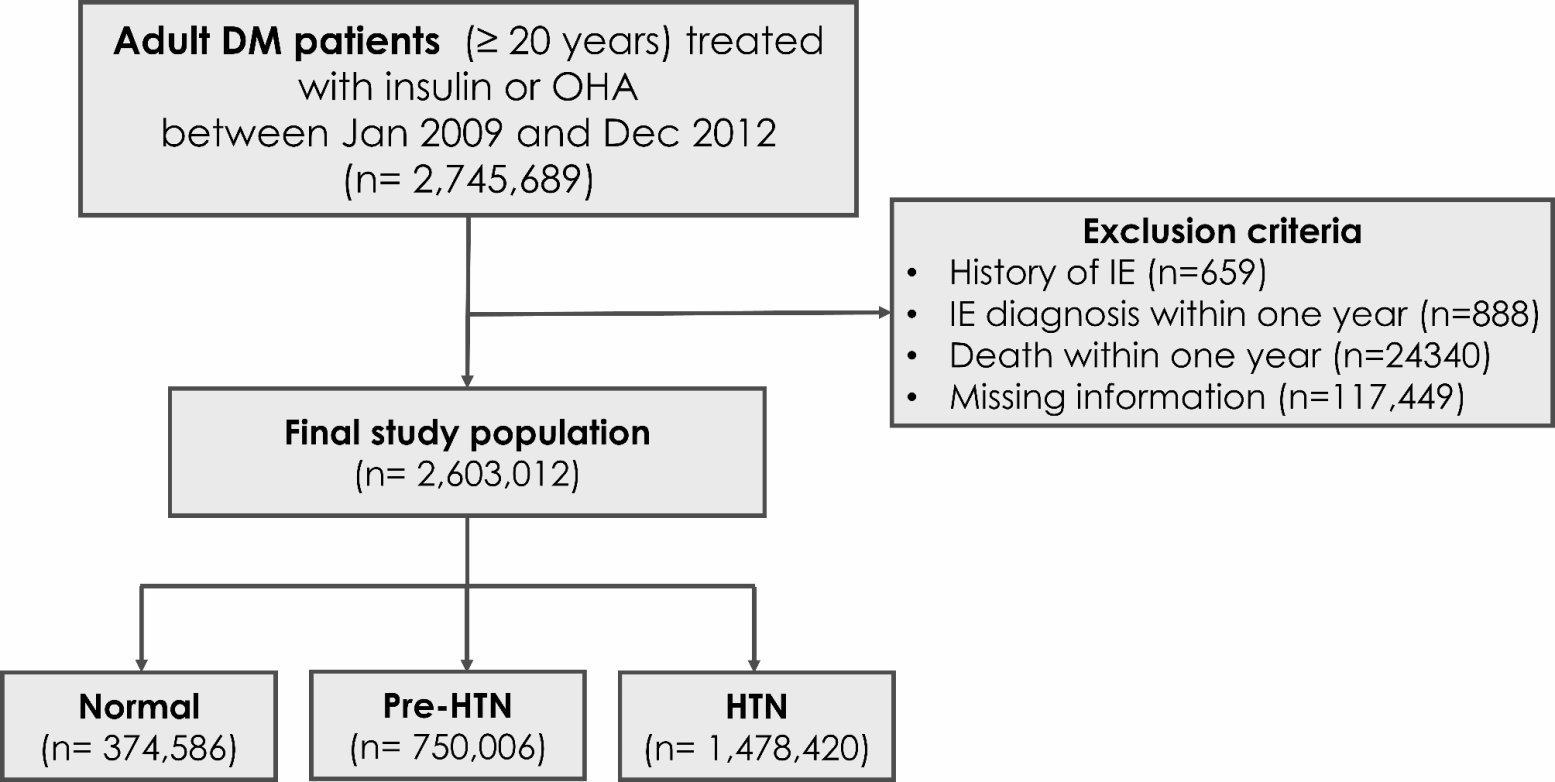
CONSORT Diagram of Study Cohort

Using this database, we initially identified 2,745,689 patients (≥ 20 years old) with DM who underwent a health examination between January 2009 and December 2012. We excluded patients with a history of IE (*n* = 659) since the current study focused on the incidence of IE, and those with IE diagnosis (*n* = 229) or died (*n* = 24,340) within one year after the day of their health screening. Patients with missing information (*n* = 117,449) were excluded as well (Fig. 1). This study was approved by the institutional review board of Soongsil University (Approval no. SSU-202003-HR-201-01).

### Definition of diabetes

The presence of DM was diagnosed if [1] *International Statistical Classification of Disease and Related Health Problems*,* Tenth Revision* (ICD-10) codes for diabetes (E11-E14) were claimed with at least one prescription of hypoglycemic agents or insulin before the health screening or [2] fasting blood glucose exceeded 126 mg/dL at the health screening.

### Exposure: hypertension and underweight

HTN was diagnosed if *ICD-10* codes for HTN (I10-I13 and I15) were claimed, and antihypertensive agents were prescribed within six months of nationwide health examination. The participants were categorized by ordinal variables as follows: normotension (systolic blood pressure [SBP] < 120 mmHg and diastolic blood pressure [DBP] < 80mmg), prehypertension (120 ≤ SBP < 140 mmHg and 80 ≤ DBP < 90 mmHg) and hypertension (SBP ≥ 140mmHg, DBP ≥ 90mmHg). Participants diagnosed with hypertension were classified into the hypertension (HTN) group, irrespective of the blood pressure measured.

Body mass index (BMI) was calculated as weight in kilograms divided by the square of height in meters (kg/m^2^). Following the World Health Organization recommendations for Asians, subjects with a BMI lower than 18.5 kg/m^2^ were defined as underweight.

### Outcome: incident IE

The study population was followed up from the date of health screening to the date of incident IE diagnosis, censoring (e.g., death), or the end of the study period (December 31, 2018), whichever came first. The primary study of interest was a new diagnosis of IE, which was defined using the ICD-10 codes for IE (I33.x, I38.x, and I39.8). Of the patients who conform to ICD codes of IE, only those who died within 14 days or were hospitalized longer than 14 days were included in the present study.

### Covariates and definition

Information regarding general health behaviors was obtained by a self-completed questionnaire. Smoking status was classified as a non-smoker (< 100 cigarettes in a lifetime), ex-smoker (smoked at least 100 cigarettes in a lifetime but had not smoked within one month of national health check-up), or current smoker (smoked at least 100 cigarettes in a lifetime and continued smoking within one month of the national health check-up). Alcohol assumption status was categorized into three groups as follows: non-drinker (0 g of alcohol per week), mild-drinker (0 g < alcohol < 210 g per week), or heavy-drinker (≥ 210 g of alcohol per week). Regular exercise was defined as either vigorous exercise of at least 20 min more than three times per week or moderate intensity exercise of at least 30 min five times per week. Regarding the comorbidities, the presence of dyslipidemia was determined if [1] the *ICD-10* code (E78) was claimed with a prescription of an antihyperlipidemic agent or [2] total cholesterol was ≥ 240 mg/dL. Glomerular filtration rate less than 60mL/min/1·73m^2^, as estimated by the Modification of Diet in Renal Disease equation, was the diagnostic criteria for chronic kidney disease (CKD). Blood samples were obtained after an overnight fast to measure serum glucose and lipid parameters, including total cholesterol and low- and high-density lipoprotein cholesterol. The low income level was defined as the bottom 20% of the house income level.

### Statistical analysis

The categorical variables were expressed as frequencies and percentages and were compared with the Chi-square or Fisher’s exact tests. The continuous variables were presented as mean ± standard deviation or median with range and were compared using Student’s *t*-test and Mann-Whitney U test as appropriate. Incidence rates were estimated using the total number of IE events during the follow-up period divided by 1000 person-years at risk. Kaplan-Meier plots were constructed to delineate the cumulative incidence of IE, and the log-rank test was used to compare the intergroup differences in the Kaplan-Meier estimates. A multivariable-adjusted Cox proportional hazards model and a competing risk model were employed to evaluate the association between HTN/ underweight and the incidence of IE. The subdistribution hazard ratio (SHR) was estimated using the Fine and Gray method, which consider death a competing risk. Age, sex, economic status, smoking, drinking, regular exercise, dyslipidemia, chronic kidney disease, diabetes duration, insulin use, and oral hypoglycemic agent medications were adjusted in the multivariable models. Stratified analyses of the association of HTN status of incident IE by age, sex, and habitual patterns were conducted. The risk of IE according to DM duration and treatment was analyzed as well. All significant tests were two-sided, and *p*-values < 0.05 were considered statistically significant. SAS statistical software version 9·4 (SAS Institute, Cary, NC) was used for statistical analyses.

## Results

### Baseline characteristics

The final study population included 2,603,012 patients, with a mean age of 57.4 ± 12.3 years, and Normal, Pre-HTN and HTN group consisted of 374,586 (14·4%), 750,006 (28·8%) and 1,478,420 (56.8%), respectively (Fig. 1). Table 1 summarizes the baseline characteristics of the study population according to HTN status. Patients in the HTN group were older, had a higher prevalence of females, and were more likely to have higher BMI, CKD, insulin therapy, and use of three or more oral hypoglycemic agents compared to the reference group. The three groups differed significantly across various parameters listed in Table 1 (all *p* < 0.001).

**Table 1:** Baseline profile

Variables, *n* (%)	Normal	Pre-HTN	HTN	*P* value
	(*n*=374,586)	(*n*=750,006)	(*n*=1,478,420)	
Age, years	52·8±12·1	53·0±12·3	60·9±11·3	<0·0001
Age				<0·0001
<40	46,832(12·5)	101,870(13·6)	47,946(3·2)	
40-64	263,953(70·5)	509,356(67·9)	847,446(57·3)	
≥65	63,801(17·0)	138,780(18·5)	583,028(39·4)	
Sex				<0·001
Male	213,800(57·1)	501,997(66·9)	846,875(57·3)	
Female	160,786(42·9)	248,009(33·1)	631,545(42·7)	
BMI, kg/m^2^	23·7±3·1	24·8±3·3	25·6±3·4	<0·001
BMI level,				<0·001
<18.5	13,243(3·5)	12,396(1·7)	15,979(1·1)	
<23	141,932(37·9)	204,318(27·2)	302,093(20·4)	
<25	98,228(26·2)	194,702(26·0)	352,609(23·9)	
<30	109,479(29·2)	292,413(39·0)	664,679(45·0)	
≥30	11,704(3·1)	46,177(6·2)	143,060(9·7)	
Smoking				<0·001
Non-smoker	206,036(55·0)	368,567(49·1)	873,831(59·1)	
Ex-smoker	58,207(15·5)	138,849(18·5)	284,087(19·2)	
Current-smoker	110,343(29·5)	242,590(32·4)	320,502(21·7)	
Drinking				<0·001
Non-drinker	223,487(59·7)	378,963(50·5)	889,328(60·2)	
Mild-drinker	123,548(33·0)	286,664(38·2)	442,002(29·9)	
Heavy-drinker	27,551(7·4)	84,379(11·3)	147,090(10·0)	
Regular exercise	75,250(20·1)	151,455(20·2)	309,411(20·9)	<0·001
Chronic kidney disease	23,681(6·3)	48,066(6·4)	229,487(15·5)	<0·001
GFR, mL/min/1.73m^2^	88·7±35·7	88·6±36·2	82·1±36·2	<0·001
DM Duration, ≥5 years	98,278(26·2)	168,939(22·5)	542,560(36·7)	<0·001
DM treatment				
Insulin	29,287(7·8)	41,622(5·6)	157,194(10·6)	<0·001
OHA, ≥3	50,091(13·4)	80,709(10·8)	246,596(16·7)	<0·001
Fasting glucose, mg/dL	147·9±49·7	149·6±47·6	141·5±45·8	<0·001
Waist circumference, cm	81·6±8·4	84·5±8·3	86·9±8·6	<0·001
Systolic BP, mmHg	109·7±7·0	127·0±6·9	135·0±16·5	<0·001
Diastolic BP, mmHg	68·5±6·0	78·5±5·9	82·0±11·1	<0·001
Dyslipidemia	123,114(32·9)	251,699(33·6)	716,824(48·5)	<0·001
Total cholesterol, mg/dL	195·4±41·6	201·7±42·1	194·2±43·2	<0·001
HDL -C, mg/dL	52·7±23·5	52·4±23·2	51·7±24·2	<0·001
LDL -C, mg/dL	113·2±39·4	114·9±41·1	108·6±41·4	<0·001
Income, low 20%	79,140(21·1)	152,459(20·3)	314,996(21·3)	<0·001

Values are n (%), mean ± standard deviation, or median [quartile 1- quartile 3]

BP, blood pressure; BMI, body mass index; DM, diabetes mellitus; eGFR, glomerular filtration rate; HDL-C, high density lipoprotein cholesterol; LDL-C, low density lipoprotein cholesterol; OHA, oral hypoglycemic agent

### Impact of hypertension on IE

**Fig. 2:**
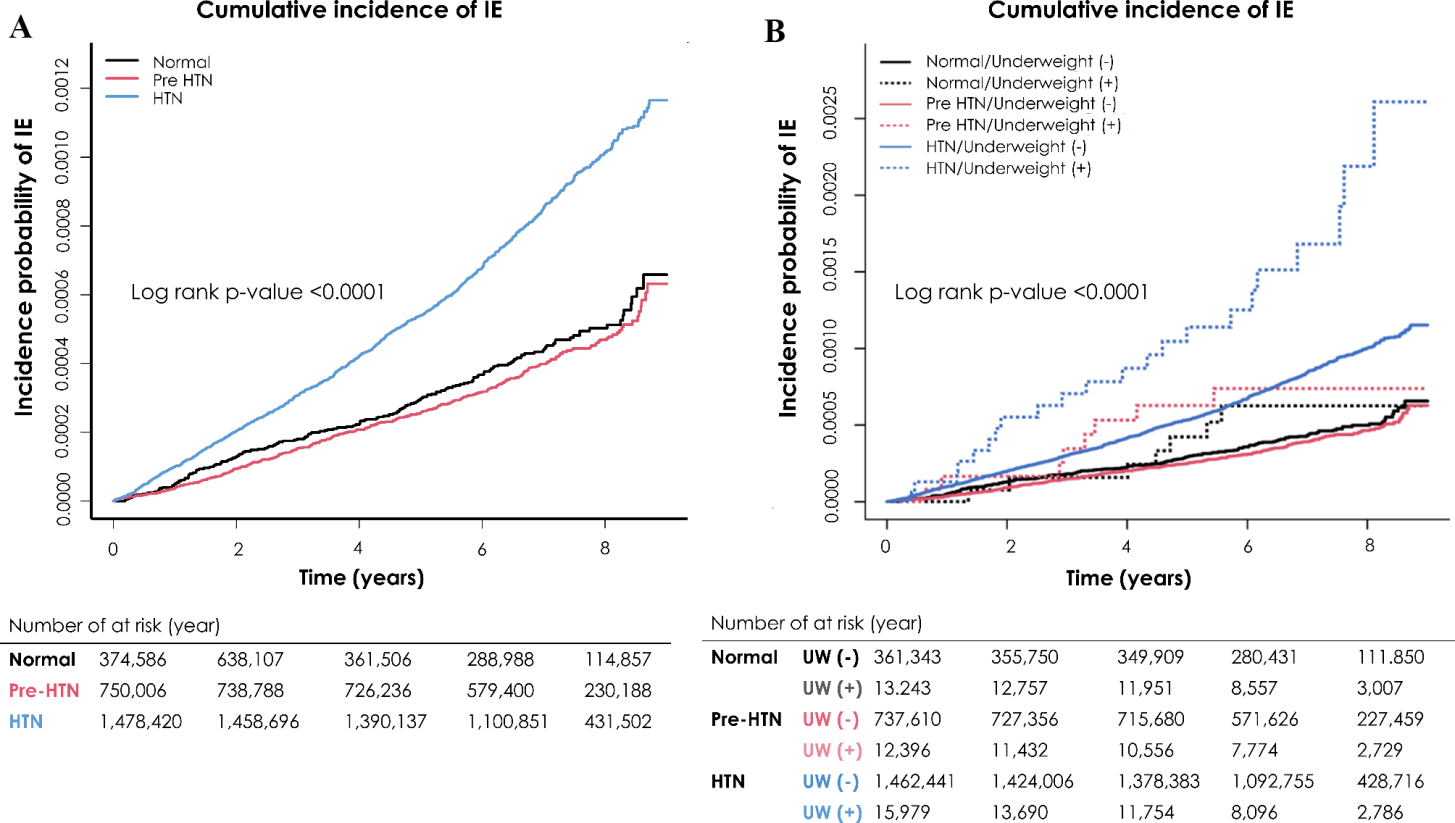
Cumulative Incidence of Infective Endocarditis. Kaplan-Meier estimates of cumulative incidence of infective endocarditis during the follow-up period were compared between the groups classified according to (**A**) HTN status, and (**B**) HTN status and presence of underweight

During the follow-up of 7.14 years (interquartile range 1–3, 6.01–8.08 years), a total of 1703 cases of IE were diagnosed. The incidence of IE in the Normal, Pre-HTN and HTN groups was 0.065, 0.058 and 0.122 person per 1000 person-years, respectively. The patients with HTN presented a 1.36-fold increased risk of IE compared to the normal reference group (SHR, 1.36; 95% confidence interval [CI], 1.152–1.607). However, no difference in the risk of IE was found between pre-hypertensive and normotensive participants (Table 2). The Kaplan-Meier plot demonstrated the cumulative hazard of IE according to hypertension status, showing that the HTN group had the highest incidence of IE among all groups (log-rank *p* < 0.001) (Fig. 2).

**Table 2:** Incidence and risk of incident IE

							HR (95% CI)	SHR(95% CI)
HTN level	UW	Participants, No.	Event, No.	Person-years, No.	IR per 1000 PY	Model 1^a^	Model 2^b^	Model 3^c^
Normal		374,586	168	2,596,618.93	0.0647	1 (Reference)	1 (Reference)	1 (Reference)
Pre-HTN		750,006	303	5,191,772.99	0.05836	0.903 (0.748-1.091)	0.909 (0.752-1.097)	0.918 (0.759- 1.109)
HTN		1,478,420	1,232	10,069,290.54	0.12235	1.892 (1.610-2.223)	1.343 (1.138-1.585)	1.360 (1.152-1.607)
							**HR (95% CI)**	**SHR(95% CI)**
Normal	No	361,343	161	2,513,483·48	0.06405	1 (Reference)	1 (Reference)	1 (Reference)
	Yes	13,243	7	83,135·44	0.0842	1.334 (0.626-2.843)	1.180 (0.554-2.517)	1.028 (0.482-2.194)
Pre-HTN	No	737,610	295	5,115,472·1	0.05767	0.902 (0.744-1.093)	0.906 (0.747-1.098)	0.913 (0.753-1.107)
	Yes	12,396	8	76,300·89	0.10485	1.663 (0.818-3.383)	1.387 (0.681-2.825)	1.209 (0.593-2.465)
HTN	No	1,462,441	1210	9981453.39	0.12122	1.894 (1.607-2.232)	1.344 (1.135-1.590)	1.357 (1.145-1.609)
	Yes	15,979	22	87,837·15	0.52046	4.012 (2.570-6.264)	2.221 (1.418-3.481)	1.748 (1.108-2.755)

^a^ Model 1 was unadjusted

^b^ Model 2 was adjusted for age, sex, lower income (lowest Q1), smoking, drinking, regular exercise, diabetes duration, chronic kidney disease, dyslipidemia, insulin use and oral hypoglycemic agents (≥3 types)

^c^ Model 3 was adjusted for age, sex, lower income (lowest Q1), smoking, drinking, regular exercise, diabetes duration, chronic kidney disease, dyslipidemia, insulin use and oral hypoglycemic agents (≥3 types), considering death as a competing event

CI, confidence interval; HR, hazard ratio; HTN, hypertension; IR, incidence rate; IE, infective endocarditis; SHR, subdistribution hazard ratio; UW, underweight

### Composite impact of hypertension and underweight on IE

The influence of HTN on the risk of IE was maintained in both patients with (SHR, 1.748; 95% CI, 1.108–2.755) and without (SHR, 1.357; 95% CI, 1.145–1.609) underweight status (Table 2). Being underweight did not significantly increase the risk of IE in the normotensive or pre-HTN diabetic cohort (Table 2). However, participants being underweight concomitant with HTN (SHR, 1.748; 95% CI, 1.108–2.755) exhibited a higher risk of IE compared to those solely with HTN (SHR, 1.357; 95% CI, 0.145–1.609).

### Subgroup analysis

**Fig. 3:**
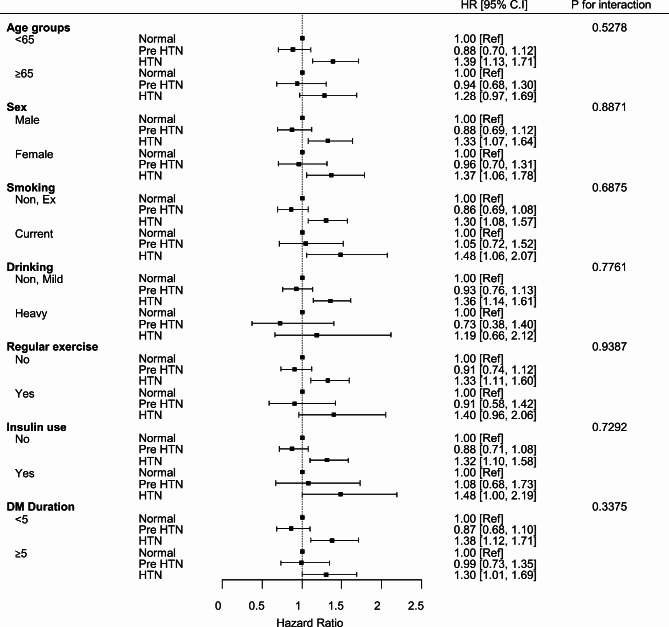
Subgroup Analysis for Incidence of Infective Endocarditis. Forest-plot summarizing the associated risk of infective endocarditis in the subgroup of age, sex, smoking, drinking, regular exercise, insulin use and duration of diabetes mellitus

In the subgroup analysis for incidence of IE among diabetic patients, there was no interaction observed with a duration of DM (< 5 years vs. ≥ 5 years), insulin use, age (< 65 years vs. ≥ 65years, sex or habitual patterns including smoking, drinking and regular exercise (Fig. 3).

## Discussion

The present study demonstrates that HTN is an independent risk factor for incident IE in people with diabetes. In addition, being underweight was associated with an increased risk of incident IE in those with concomitant HTN.

A higher risk of various infectious diseases, including IE, in diabetic individuals than non-diabetic people has been shown in several series [7, 9, 17, 18]. A population-based study conducted in Spain showed that people with type 2 DM had more than twice the incidence of IE than those without DM [8]. Furthermore, a stepwise increase in the risk of IE with DM duration was indicated in the cited study using Danish nationwide registries [19]. However, there is an unmet need for risk analysis of incident IE in the diabetic population, which may provide a chance to prevent and improve the management of IE.

In a recent publication using national cohort data, Lee et al. demonstrated that increased blood pressure was associated with an increased risk for IE in a dose-dependent manner [12]. In line with this, our study showed that HTN was an independent predictor of incident IE in the diabetic cohort as well. However, unlike DM, which is known to cause immune dysfunction, angiopathy, or gastrointestinal and urinary dysmotility, the mechanism by which HTN causes incident IE is still unclear [10]. One of possible suggestion is a high shear stress applied on the valve leaflets, and another is the cardiac valvular endothelial injury aggravating secondary atherosclerosis and calcification [20–22]. These pathologic changes may further predispose diabetic patients to IE.

We also found that being underweight concomitant to HTN was a secondary risk factor for incident IE. Cited studies showed that being underweight was associated with a higher risk of infection than obesity, and diabetic people who were underweight were more likely to have a poorer prognosis in the treatment of infectious disease compared to those with obesity, so-called obesity paradox [9, 23, 24]. In our analysis, although underweight was not identified as an independent risk factor for IE, it exacerbated the risk of incident IE in diabetic patients with HTN. This finding is in line with previous diabetes studies demonstrating adverse outcomes in underweight individuals compared to those with overweight.

### Limitations

The present study has several limitations to be acknowledged. First, the study contains inherent limitations of retrospective design. Second, the direct causality between HTN and incident IE was not established in the current study. Additionally, since hypertension was diagnosed using *ICD-10* codes in the present study, there is a potential for misclassification in patients who have been prescribed angiotensin receptor blockers due to proteinuria. Finally, the study results have limited applicability to other ethnic groups as the study cohort was composed mostly of Korean diabetic populations.

## Conclusion

In the diabetic population, HTN played a role as a predictor of incident IE. Moreover, being underweight may cause those with concomitant HTN to be more susceptible to IE. Therefore, a rigorous and timely management of HTN and simultaneous avoidance of excessive weight loss may be required to prevent the incident IE in the diabetic population.

## Data Availability

The data that supported the findings of the present study are available from the K-NHIS. However, restrictions apply regarding the availability of the data, which were used with permission for this specific study.

## Abbreviations

CKD: Chronic Kidney Disease
CI: Confidence Interval
DM: Diabetes Mellitus
DBP: Diastolic Blood Pressure
HR: Hazard Ratio
HTN: Hypertension
IE: Infective Endocarditis
ICD: International Statistical Classification of Disease and Related Health Problems
SBP: Systolic Blood Pressure
